# Effects of Ethanol and NAP on Cerebellar Expression of the Neural Cell Adhesion Molecule L1

**DOI:** 10.1371/journal.pone.0024364

**Published:** 2011-09-08

**Authors:** Devon M. Fitzgerald, Michael E. Charness, Kimberly A. Leite-Morris, Suzhen Chen

**Affiliations:** 1 Veterans Affairs Boston Healthcare System, Boston, Massachusetts, United States of America; 2 Department of Neurology, Harvard Medical School, West Roxbury, Massachusetts, United States of America; 3 Boston University School of Medicine, Boston, Massachusetts, United States of America; 4 Departments of Psychiatry, Pharmacology and Experimental Therapeutics, Boston University School of Medicine, Boston, Massachusetts, United States of America; University of Memphis, United States of America

## Abstract

The neural cell adhesion molecule L1 is critical for brain development and plays a role in learning and memory in the adult. Ethanol inhibits L1-mediated cell adhesion and neurite outgrowth in cerebellar granule neurons (CGNs), and these actions might underlie the cerebellar dysmorphology of fetal alcohol spectrum disorders. The peptide NAP potently blocks ethanol inhibition of L1 adhesion and prevents ethanol teratogenesis. We used quantitative RT-PCR and Western blotting of extracts of cerebellar slices, CGNs, and astrocytes from postnatal day 7 (PD7) rats to investigate whether ethanol and NAP act in part by regulating the expression of L1. Treatment of cerebellar slices with 20 mM ethanol, 10^−12^ M NAP, or both for 4 hours, 24 hours, and 10 days did not significantly affect L1 mRNA and protein levels. Similar treatment for 4 or 24 hours did not regulate L1 expression in primary cultures of CGNs and astrocytes, the predominant cerebellar cell types. Because ethanol also damages the adult cerebellum, we studied the effects of chronic ethanol exposure in adult rats. One year of binge drinking did not alter L1 gene and protein expression in extracts from whole cerebellum. Thus, ethanol does not alter L1 expression in the developing or adult cerebellum; more likely, ethanol disrupts L1 function by modifying its conformation and signaling. Likewise, NAP antagonizes the actions of ethanol without altering L1 expression.

## Introduction

The L1 neural cell adhesion molecule is critical for brain development. L1 mediates cell-cell interactions, neuronal migration, neurite outgrowth, axon guidance and fasciculation, and neuronal survival in the developing nervous system [1]. L1 expression persists in the adult nervous system, where it is believed to play a role in learning, memory, and regeneration after injury [2]–[5]. L1 gene mutations cause a spectrum of dysmorphic lesions, including hydrocephalus, agenesis or hypoplasia of the corpus callosum, and cerebellar dysplasia, referred to as CRASH or L1 syndrome [6], [7]. The similarity of the lesions of L1 syndrome to those of fetal alcohol spectrum disorders (FASD) led to the hypothesis that ethanol causes FASD in part by disrupting L1-mediated processes [8], [9]. In support of this hypothesis, concentrations of ethanol attained after one drink inhibit L1-mediated cell-cell adhesion (L1 adhesion) in transfected fibroblasts, neural cell lines, and cerebellar granule neurons (CGNs) [8]–[10]. Furthermore, ethanol inhibits L1-mediated neurite outgrowth in CGNs at similarly low concentrations [11]. Finally, drugs that block ethanol inhibition of L1 adhesion also prevent ethanol teratogenesis in mouse embryos [12]–[15]. One such ethanol antagonist, the peptide NAPVSIPQ (NAP), blocks ethanol inhibition of L1 adhesion at femtomolar concentrations [16].

Several mechanisms might account for how ethanol disrupts L1 function. Recent data suggest that ethanol alters extracellular domain interactions that are critical for L1 homophilic binding [17], [18]. Ethanol also disrupts L1 activation of intracellular signaling events [19], [20]. It is unknown whether regulation of L1 expression also contributes to ethanol neurotoxicity. Reductions in L1 expression could not occur rapidly enough to account for acute ethanol inhibition of L1 adhesion; however, changes in L1 expression after longer periods of ethanol exposure would disrupt both L1 adhesion and L1-mediated neurite outgrowth. Furthermore, NAP-induced up-regulation of L1 expression could partly compensate for ethanol inhibition of L1 adhesion.

Ethanol damages the developing and adult cerebellum [21]–[23]. Because L1 is critical for cerebellar development and survival of cerebellar neurons [1], [24], ethanol could damage the cerebellum by altering the expression of L1. Indeed, another teratogen, polychlorinated biphenyls (PCBs), significantly reduced L1 expression in whole cerebellum [25]. The effects of ethanol on L1 expression have not been well studied. Chronic ethanol treatment did not reduce L1 protein expression in the NG108-15 neural cell line [9] and transiently increased L1 gene expression in B104 neuroblastoma cells [26]. However, it is unknown whether ethanol modulates the expression of L1 in cerebellum, nor whether NAP antagonizes ethanol inhibition of L1 function by increasing L1 expression.

We systematically investigated the effects of ethanol and NAP exposure on L1 mRNA and protein expression in cerebellar slices, CGNs, and astrocytes of postnatal day 7 (PD7) rats. Vulnerability to binge alcohol-induced cerebellum damage is greatest during PD4-PD9 in rats, the period that corresponds to gestational weeks 24–32 in humans [27], [28]. At this developmental stage, cerebellar neurons undergo neuritogenesis and express high levels of L1 [2], [29]. Because alcoholics frequently develop cerebellar degeneration [23], [30], we also examined the effects of long-term binge drinking on L1 expression in adult rat cerebellum. Here we present evidence that ethanol does not regulate L1 expression in the developing or adult cerebellum. Similarly, NAP or the combination of ethanol and NAP do not alter L1 mRNA or protein levels in the developing cerebellum.

## Results

### Quality control, assay reliability, and validation of endogenous controls

**Figure 1:**
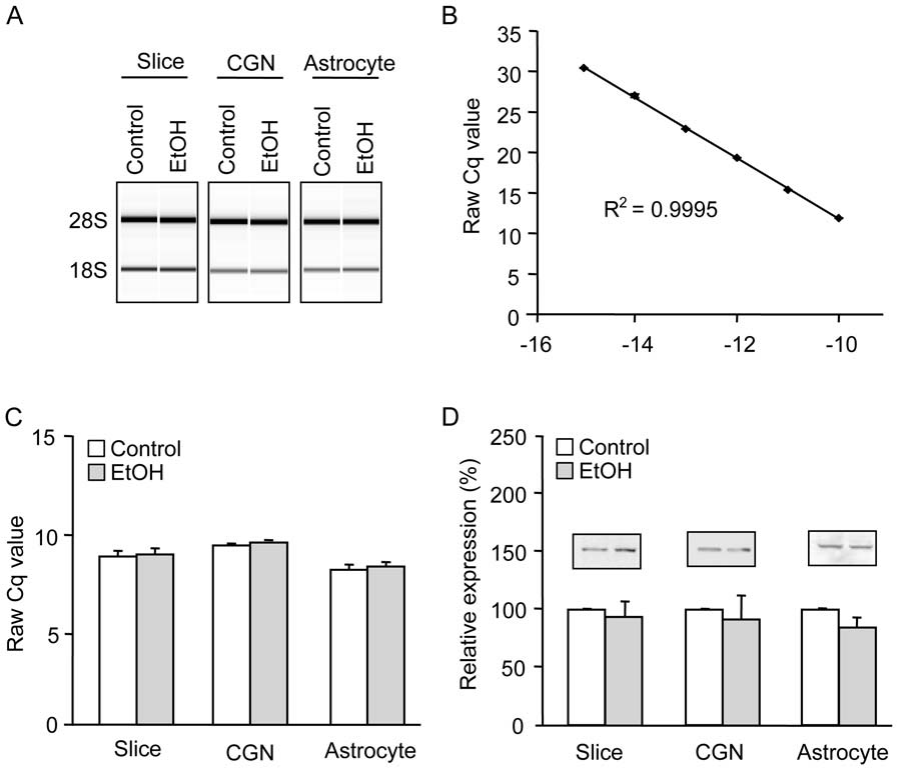
RNA quality, assay reliability, and validation of endogenous controls. Total RNA quality was assessed using an Agilent Bioanalyzer 2100 and endogenous controls were validated for RT-qPCR and Western blot. A) Generated gel images show representative slice, CGN, and astrocyte samples with and without 24-hr ethanol treatment. B) A standard curve was performed using a dilution series of purified L1 template. Increases in template concentration result in decreasing Cq values, as expected for a well-functioning qPCR assay (n = 4). C) 18S Cq values are shown for all tissue/cell types with and without ethanol treatment. D) Representative Western blots for β-tubulin are shown for each sample type with and without 24-hr ethanol treatment. All bars (C, D) show the mean + SEM of 4 independent experiments.

High-quality RNA preparations are necessary to insure that measured quantities of gene transcripts are representative of *in vivo* expression levels [31]. Therefore, the 28S:18S ribosomal RNA (rRNA) ratios, RNA integrity numbers (RINs), and yield were measured for every RNA sample prior to use in quantitative reverse transcription PCR (qRT-PCR). All samples had an RIN above 8.5 (data not shown), and treatment with ethanol did not degrade RNA quality (Fig. 1A). Average quantification cycle values (Cq) were linear (R^2^ = 0.9995) with log-transformed L1 transcript concentration over at least six log orders (Fig. 1B). Average quantification cycle (Cq) values from control and ethanol-treated samples were used to evaluate the stability of potential endogenous control genes, and 18S rRNA (18S) was found to be more stable than cyclophilin A (not shown) in all cell and tissue types (Fig. 1C). Due to its stability with ethanol treatment, 18S was used to normalize all L1 mRNA expression data. Similarly, we evaluated the effects of ethanol on levels of expression of candidate endogenous protein controls, including β-tubulin (Fig. 1D), glyceraldehyde 3-phosphate dehydrogenase (GAPDH), and actin (not shown). Among these, only β-tubulin protein expression was unaffected by ethanol treatment in all three culture preparations.

### Effects of ethanol and NAP on L1 expression in early postnatal cerebellum

**Figure 2:**
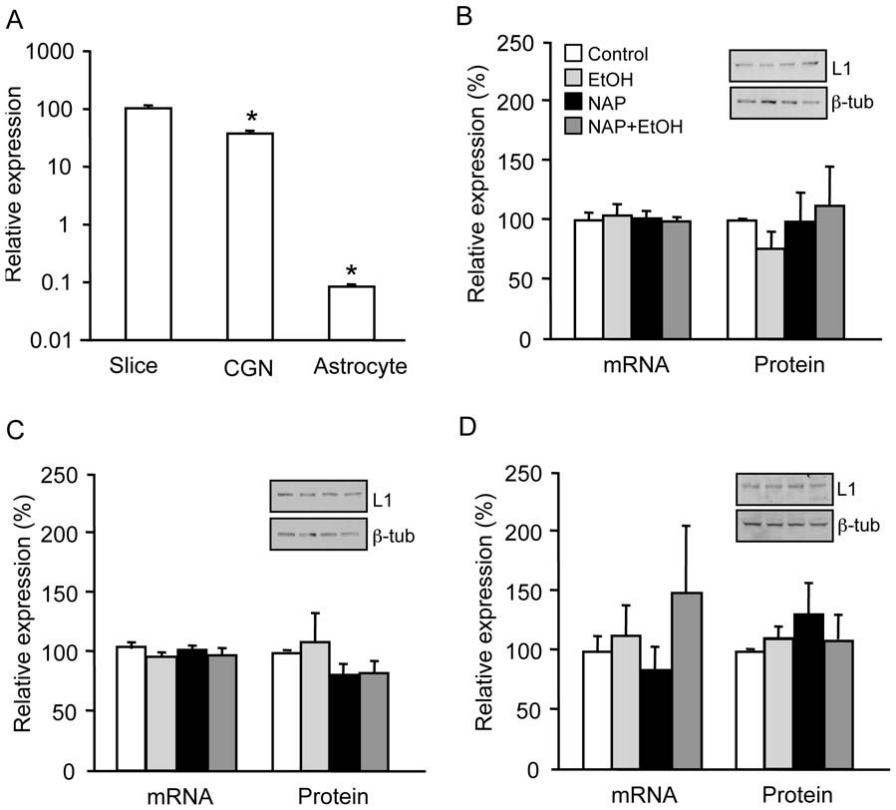
L1 expression in cerebellar slices, CGNs, and astrocytes. A) Comparison of L1 transcript levels in different culture preparations. * p<0.0001 compared to slices. B–D) Total RNA and cell lysates were collected from slice, CGN, and astrocyte cultures after 24-hr exposure to 20 mM ethanol, 10^−12^ M NAP, or the combination of NAP and ethanol. L1 mRNA expression was measured by qRT-PCR and normalized to 18S. L1 protein levels were measured by Western blot and normalized to β-tubulin (representative images shown above corresponding bars). L1 mRNA and protein levels are shown in cerebellar slices (B), CGNs (C), and astrocytes (D). Legend in B applies to B–D. All bars represent normalized mean + SEM of 4 independent experiments.

We first determined relative levels of L1 mRNA in the different culture preparations. L1 expression was 62.1±4.2% lower in CGNs than in slices and 99.9±0.01% lower in astrocytes than in slices (n = 4; p<0.0001 for each comparison)(Fig. 2A). To determine the effects of ethanol and NAP on L1 expression, cerebellar slices from PD7 rats were exposed to 20 mM ethanol, 10^−12^ M NAP, or both for 4 hours, 24 hours, or 10 days. Treatment with ethanol or NAP had no effect on levels of L1 mRNA or protein at any of the time points (Fig. 2B, Table 1). Treatment with ethanol and NAP for 10 days significantly reduced L1 mRNA, but had no significant effect on L1 total protein expression (Table 1).

**Table 1:** Summary of ethanol and NAP effects on L1 mRNA and protein expression in three cerebellar culture systems.

Sample type	L1 mRNA (% RQ±SEM)	n	p	L1 protein (% RQ±SEM)	n	p
***Cerebellar slices***						
***4 hr***						
Control	100±13	7	-	100	6	-
EtOH	102±9	7	0.92	82±16	6	0.30
NAP	91±4	4	0.59	121±59	6	0.73
NAP+EtOH	114±9	4	0.46	88±21	6	0.60
***24 hr***						
Control	100±6	4	-	100	4	-
EtOH	104±10	4	0.83	76±14	4	0.19
NAP	101±7	4	0.91	98±26	4	0.94
NAP+EtOH	99±4	4	0.85	112±33	4	0.75
***10 day***						
Control	100±10	4	-	100	3	-
EtOH	108±8	4	0.45	87±40	3	0.80
NAP	97±3	4	0.94	100±28	3	0.99
NAP+EtOH	65±7	4	*0.03*	113±42	3	0.78
***CGNs***						
***4 hr***						
Control	100±12	5	-	100	4	-
EtOH	115±13	5	0.39	109±19	4	0.66
NAP	118±6	5	0.24	92±10	4	0.50
NAP+EtOH	131±7	5	0.07	99±32	4	0.98
***24 hr***						
Control	105±6	4	-	100	4	-
EtOH	96±4	4	0.25	109±25	4	0.76
NAP	103±4	4	0.77	81±10	4	0.16
NAP+EtOH	98±7	4	0.48	83±11	4	0.22
***Astrocytes***						
***4 hr***						
Control	100±10	3	-			
EtOH	85±13	3	0.47			
NAP	77±11	3	0.20			
NAP+EtOH	97±15	3	0.90			
***24 hr***						
Control	100±15	8	-	100	4	-
EtOH	114±26	6	0.63	111±10	4	0.37
NAP	84±20	5	0.52	132±26	4	0.31
NAP+EtOH	150±57	3	0.23	109±22	4	0.72

Cerebellar slices comprise a mixture of cell types, so experiments on slices might obscure opposite effects on different cell types. Therefore, we conducted separate experiments on primary cultures of CGNs and astrocytes, the predominant cell types of the cerebellum. In CGNs, treatment with ethanol, NAP, or both for 4 or 24 hours had no significant effect on the expression of L1 mRNA or protein (Fig. 2C, Table 1). L1 expression was significantly lower in astrocytes compared with CGNs, as previously described [32], but also showed no significant changes with ethanol, NAP, or combined treatments (Fig. 2A,D). Higher concentrations of ethanol (100 mM) likewise had no effect on L1 expression in cerebellar slices, CGNs, and astrocytes (not shown).

### Effects of ethanol and NAP on L1 expression in adult cerebellum

**Figure 3:**
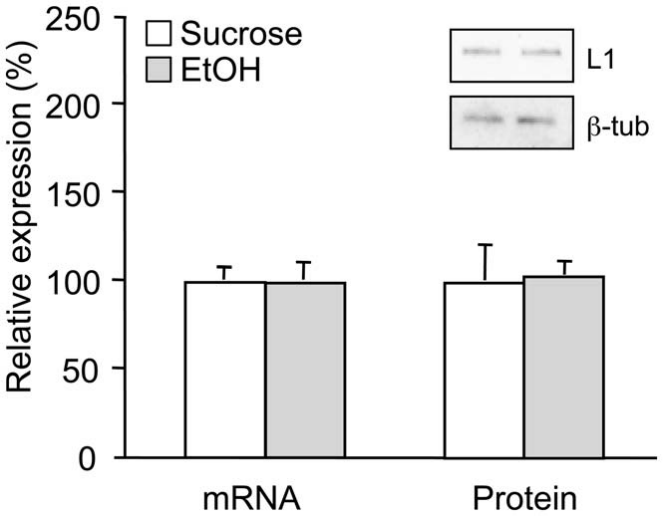
L1 expression in adult cerebellum after chronic binge drinking. Total RNA and tissue lysates were collected from cerebella of adult rats following one year of self-administration of ethanol (2% sucrose/10% ethanol) or sucrose (2% sucrose). L1 mRNA expression was measured by qRT-PCR and normalized to 18S. L1 protein levels were measured by Western blot and normalized to β-tubulin. The inset shows representative Western blot images above corresponding bars. Bars represent normalized mean + SEM (ethanol, n = 6; sucrose, n = 7).

We used a chronic binge-drinking rat model [33] to evaluate the effects of ethanol on L1 expression in adult cerebellum. We measured L1 mRNA and protein expression in whole cerebellar homogenates from rats that had self-administered ethanol for more than 12 months, beginning at approximately 2 months of age. Subjects that self-administered ethanol attained mean blood ethanol concentrations of 100±14 mg/dl (n = 6). Cerebellar L1 mRNA and protein levels did not differ between ethanol-exposed rats (n = 6) and sucrose controls (n = 7)(Fig. 3).

## Discussion

The major finding of this work is that ethanol does not regulate L1 gene or protein expression in the developing or adult cerebellum. Likewise, the alcohol antagonist NAP, either alone or in combination with ethanol, does not regulate L1 expression in three models of the developing cerebellum.

### Validity of the experimental results

Accurate gene and protein expression analysis requires rigorous techniques and the appropriate selection of endogenous reference genes or proteins for the experimental conditions. We followed all of the recent recommendations for the reliable measurement of mRNA by qRT-PCR [34], [35]. In particular, we isolated high-quality RNA with an RIN that was consistently higher than 8.5 - well above the recommended threshold for qRT-PCR [31], [36]. Furthermore, we fully validated our primer pairs and performed standard curves to verify PCR efficiency and exclude the presence of PCR inhibitors. Finally, we also verified that our endogenous reference standards - 18S for RNA and β-tubulin for protein – were not influenced by ethanol treatment in any of the *in vitro* model systems.

### Ethanol does not affect L1 expression in developing cerebellum

We studied PD7 rats, because the cerebellum is particularly vulnerable to ethanol exposure at this developmental time point [27], [28], when L1 plays a critical role in CGN differentiation, migration, and survival [2], [24], [37]–[40]. L1 regulates CGN migration and axon outgrowth and is a survival factor for CGNs [24], [38], [39]; therefore, ethanol-induced reductions in L1 expression could disrupt cerebellar development. We used three different model systems to evaluate the effects of ethanol exposure on L1 expression. Cerebellar slices preserve the integrity of cerebellar circuitry and neuronal-glial interactions in the developing cerebellum [41]. Cerebellar granule neurons and astrocytes are the most abundant neuronal and glial cell types, respectively, in the developing cerebellum and both show ethanol-induced cell death [42], [43]. Treatment with intoxicating (20 mM) or anesthetic (100 mM) concentrations of ethanol for 4 hours, 24 hours, or 10 days did not reduce L1 expression in cerebellar slices, CGNs, and astrocytes, with one exception. After 10 days of ethanol plus NAP treatment, there was a decrease in L1 mRNA in cerebellar slices, but this was of dubious functional significance, since there was no corresponding change in L1 protein expression.

Our failure to observe changes in L1 mRNA is not likely a consequence of the insensitivity of our assays or the unresponsiveness of our culture systems to ethanol. Our qRT-PCR assay was highly sensitive and linear in detecting differences in L1 transcript levels and allowed us to observe significant differences in L1 expression among cerebellar slices, CGNs, and astrocytes. Furthermore, the ethanol dose and duration of treatment in these experiments are sufficient to modify cerebellar physiology [44], [45], neuronal differentiation [11], [46], and gene expression [47]–[51]. Taken together, our findings suggest that ethanol does not disrupt cerebellar development by altering L1 expression.

Although previous work in CGNs demonstrated that ethanol inhibits L1 adhesion within 30 minutes [8] and L1-mediated neurite outgrowth within 12 hours [11], [19], our data indicate that neither of these effects can be attributed to ethanol-induced reductions in L1 expression. Indeed, recent studies suggest that ethanol inhibits L1 adhesion by disrupting the interactions of the Ig1 and Ig4 extracellular domains [17], [18]. Further work is required to learn whether ethanol modulates L1 expression in other cerebellar cell types, such as Purkinje cells, Golgi neurons, microglia, and oligodendrocytes. Likewise, it remains to be determined whether ethanol modulates the subcellular distribution of L1 in CGNs. Recent studies indicate that ethanol does not change the polarity of L1 sorting within dorsal root ganglion cells [19].

### NAP does not regulate L1 expression in developing cerebellum

NAP is neuroprotective against a variety of insults, including fetal alcohol exposure, although the underlying mechanisms are unclear [12], [15], [52]–[54]. Our studies were designed to evaluate whether NAP up-regulates L1 expression, which could compensate for ethanol inhibition of L1 function. Treatment of cells with NAP, alone or in combination with ethanol, had no effect on L1 protein expression at any of the time points in any of the *in vitro* model systems. Similar concentrations of NAP induced axon outgrowth in CGNs and blocked ethanol-induced teratogenesis in mouse embryos [12], [46]. Therefore, it is unlikely that NAP prevents ethanol teratogenesis by regulating the expression of L1. In addition to blocking ethanol inhibition of L1 adhesion, NAP might prevent ethanol teratogenesis by blocking ethanol-induced decreases in levels of reduced glutathione and in GABA_A_β3 receptor and BDNF gene expression [15], [55], [56].

### Ethanol does not regulate L1 expression in adult cerebellum

Cerebellar atrophy is a common finding in alcoholics in both imaging and autopsy studies [57], [58]. Because L1 is a neuronal survival factor, ethanol effects on L1 expression could mediate alcoholic cerebellar degeneration. Our data demonstrate that one year of binge drinking to intoxicating blood ethanol concentrations did not alter L1 gene and protein expression in adult cerebellum. These findings make it less likely that ethanol causes cerebellar degeneration in part by down-regulating L1 expression. Further studies with other alcohol exposure paradigms and in other species would strengthen this conclusion.

## Materials and Methods

### Materials

Sprague Dawley rats from Charles River Laboratories (Wilmington, MA) were used for all cell and tissue culture experiments. Mothers and pups were allowed to acclimate for at least 24 hr prior to sacrifice. Male Long Evans rats (average age 7 weeks) were purchased from Harlan (Indianapolis, IN) and had an average body weight of 225 g at the start of training in binge-drinking experiments. Subjects were allowed to acclimate to the new environment for 5 days prior to any treatment. All animals were maintained on a light/dark cycle (0600 h to 1800 h) with access to food and water *ad libitum*. Animal care procedures were conducted in accordance with NIH guidelines and the approval of the Institutional Animal Care and Use Committees at the VA Boston Healthcare System and Boston University School of Medicine.

Neurobasal medium, Dulbecco's Modified Eagle Medium (DMEM), Hank's Balanced Salt Solution (HBSS), horse serum, bovine serum, Penicillin-Streptomycin-L-Glutamine (PSG), Penicillin-streptomycin (PS), HEPES buffer, and L-glutamine were acquired from Gibco (Carlsbad, CA). Glasgow Minimal Essential Medium (MEM), glucose, sodium bicarbonate, human apo-transferrin, L-thyroxine, selenium selenate, bovine insulin, bovine aprotinin, albumin from bovine serum (BSA), poly-L-lysine (pLL), and anti-GFAP antibody were acquired from Sigma Aldrich (St. Louis, MO). Hyclone bovine calf serum was obtained from Thermo Scientific (Waltham, MA), trypsin and DNase were obtained from Worthington (Lakewood, NJ), and 10× HBSS was obtained from Cellgro (Manassas, VA). Millipore (Billerica, MA) Millicell cell culture inserts were used in slice culture. Ethanol (anhydrous, 200-proof) from Sigma Aldrich was used for all treatments in cell and tissue culture. In the binge drinking experiments the ethanol solution was diluted from non-denatured 200-proof ethanol obtained from Pharmaco-AAPER, (Brookfield,CT), and the sucrose solution was made with Domino® sugar. The NAP peptide (NAPVSIPQ) was synthesized by New England Peptide (Gardner, MA).

All RNA preparation reagents were obtained from Qiagen (Valencia, CA), with the exception of ethanol from Sigma Aldrich and chloroform from Shelton Scientific (Shelton, CT). The RNA Nano Chip kit was acquired from Agilent (Santa Clara, CA). All reverse transcriptase PCR and quantitative PCR reagents were obtained from Promega (Madison, WI), with the exception of target-specific primers, which were synthesized by Invitrogen (Carlsbad, CA).

Radio-immunoprecipitation assay (RIPA) buffer, SDS-Tris-Glycine running buffer, transfer buffer, Tris-buffered saline (TBS), and Tris-buffered saline with Tween 20 (TBST) were from Boston BioProducts (Ashland, MA). Bovine serum albumin (BSA) fraction V was obtained from EMD (Gibbstown, NJ), instant non-fat dry milk was purchased at a local grocery store, and methanol was purchased from Sigma Aldrich. Complete Mini EDTA-free protease inhibitor cocktail was obtained from Roche (Basel, Switzerland). HALT phosphatase inhibitor, Pierce BCA protein concentration assay kit, and Pierce ECL Western blotting substrate were purchased from Thermo Scientific. Mini-PROTEAN TGX pre-cast gels (4–15%) and Trans-Blot nitrocellulose membranes were obtained from BioRad (Hercules, CA), and Re-blot Plus was acquired from Millipore. L1 goat polyclonal primary antibody (SC1508) and rabbit polyclonal ß-tubulin antibody (SC9104) were obtained from Santa Cruz Biotechnology (Santa Cruz, CA), and all secondary antibodies were acquired from Jackson ImmunoResearch (West Grove, PA).

### Culture of cerebellar slices, CGNs, and astrocytes

All cultured cells and tissues were derived from PD7 rat cerebella. Pups were sacrificed with CO_2_, followed by cervical dislocation and decapitation. Cerebella were removed and meninges and blood vessels were dissected away in ice-cold HHGN (HBSS with 2.5 mM HEPES, 35 mM glucose, and 4 mM sodium bicarbonate).

Cerebellar slices were prepared as previously described [59]. Briefly, cerebella were cut into 350 µm slices using an 800 series McIlwain Tissue Chopper. Slices were manually separated and plated on Millicell cell culture inserts. Cultures were maintained in Glasgow MEM with 25% horse serum, 12.5 mM HEPES, 2.5% 10× HBSS, 1% PSG, and 22 mM glucose.

Primary CGN cultures were prepared as previously described [60]. Briefly, coarsely-chopped cerebella were incubated in 1% trypsin/0.05% DNase for 16 min and washed with HBSS. Cells were dissociated in 0.05% DNase solution by mechanical trituration. CGNs were separated by centrifugation (120 g) through a cushion of HBSS and Neurobasal medium with 15% bovine serum. Pelleted CGNs were washed with HBSS, followed by culture medium. Cells were plated on pLL-coated plates and maintained in Neurobasal medium supplemented with 1 mg/ml BSA, 10 µg/ml human apo-transferrin, 4 nM L-thyroxine, 30 nM selenium selenate, 1 µl/ml bovine aprotinin, 1 µg/ml insulin, and 2 µM L-glutamine.

Primary astrocytes were cultured as described [61], with modifications. Total cerebellar cells were dissociated as described for CGN culture. Cells were plated on pLL-coated plates in DMEM containing 10% Hyclone bovine calf serum and 1% PS. After approximately 10 days in culture, cells were shaken at 200 rpm for 6 hours to remove microglia and oligodendrocytes. Adherent astrocytes were maintained for further culture. Astrocyte purity was assessed using immunocytochemistry for GFAP and determined to be greater than 98% in representative cultures (data not shown).

Slices and CGNs were cultured for 24 to 48 hr before treatment. Astrocytes were maintained in culture for 3 to 8 passages (6–10 weeks) prior to treatment. All cultures were treated by refreshing medium and supplementing with 20 mM ethanol, 10^−12^ M NAP, or both.

### Chronic binge-drinking animal model

Self-administration training was conducted in operant chambers (Med-Associates, St. Albans, VT) equipped with a light, retractable lever, and retractable double ball bearing sipper to prevent leakage. All subjects were initially trained by drinking 10% sucrose for several days and then randomly divided into ethanol (n = 6) and sucrose (n = 7) groups using a modification of the sucrose fading procedure [62], [63]. Training was started on a continuous reinforcement schedule with a fixed ratio (FR1-FR4) that transitioned to a response requirement (RR4 to RR20). Subjects had access to the sipper tube daily for 20 minutes, 5 days per week and attained an average ethanol daily intake of 1.15±0.003 g/kg. Two weeks prior to the end of the experiment, blood samples were collected from snipped tails following a 20-minute drinking session. Blood ethanol concentrations were determined using an Analox GM7 Analyzer (Analox Instruments, Lunenburg, MA). After one year of drinking and two hours after the last drink, animals were sacrificed, and the cerebella were removed for mRNA and Western blot analysis.

### RNA isolation and cDNA synthesis

Total RNA was prepared using RNeasy Lipid Tissue Mini-kit, following the manufacturer's protocol. Briefly, Qiazol buffer was added to all samples. Afterwards, slices were disrupted by manual grinding and cell culture samples were lysed and suspended by scraping. All samples were sonicated for 1 min on ice to achieve complete homogenization. RNA was purified using spin columns. For chronic binge drinking animals, a portion of each cerebellum was preserved in RNAlater before processing for RNA preparation. RNA yield and quality were measured with an RNA Nano Chip kit using an Agilent Bioanalyzer 2100.

Total RNA samples were reverse-transcribed using GoScript Reverse Transcription System, following the manufacturer's protocol. Reactions were performed with 0.5 µg total RNA, 0.5 µg random hexamers, 3.75 mM MgCl_2_, 0.5 mM PCR nucleotides, 20 U RNasin, and 1 µL GoScript RT enzyme in 20 µL total volume. RNA and random hexamers were combined and incubated at 70°C for 5 min before combination with other components. Reactions were incubated at 25°C for 5 min to allow primer annealing, followed by 42°C for 1 hr for extension. The RT enzyme was inactivated by incubation at 70°C for 15 min. “No RT” reactions were performed for each sample by omitting the RT enzyme.

### Quantitative real-time PCR

Primer pairs to amplify L1, 18S, and cyclophilin A (CypA) were used directly or with modifications from previously published sequences (Table 2) [64]–[66]. The sequences were analyzed using Primer-BLAST (NCBI) to assess amplicon specificity, size, and location. To confirm primer specificity, melting curves were performed and showed a single peak for each reaction, indicating a single amplicon and no primer dimerization. Additionally, gel electrophoresis confirmed amplicon size.

**Table 2:** Primers used for PCR amplification of target genes.

Target (RefSeq)	Primer sequence	Location
**L1** (NM_017345)	**F**-GCCTGACACCAAATATGAGATCCACC	3346
	**R**-CTGACAAAGGCGATGAACCA	3489
**18S** (M11188.1)	**F**-GGACACGACAGGATTGACA	1278
	**R**-ACCCACGGATCGAGAAAGA	1327
**CypA** (NM_017101.1)	**F**-TGTGCCAGGGTGGTGACTT	224
	**R**-TCAAATTTCTCTCCGTAGATGGACTT	293

GoTaq Master Mix kits were used to amplify target genes for quantitative real time PCR (qPCR). This chemistry utilizes a SYBR-Green dye analog to bind double-stranded DNA. Reactions were performed in triplicate and each contained 1× GoTaq master mix (12.5 µL), 1× carboxy-X-rhodamine dye (0.25 µL), 100 nM forward and reverse primers, and 10 ng cDNA in 25 µL total volume. Amplification and data collection were performed in an ABI 7900 Signal Detector using a 96-well format. Cycling consisted of an initial denaturation step (95°C for 2 min), followed by 40 cycles at 95°C for 15 sec and 60°C for 1 min.

Standard curves were constructed by serially diluting purified PCR products for each gene target. Curves contained six template concentrations spanning 1 fg to 100 pg and the plots of log-transformed template concentrations against quantification cycle (Cq) values showed linear relationships with R^2^ values greater than 0.99 for each target. When calculated from these curves, the efficiencies of L1 and 18S amplification were 85% and 89%, respectively. All experimental Cq values fell within the range of the standard curves, insuring that they were above the limit of detection and within the linear dynamic range. A similar dilution series was prepared with pooled slice, CGN, and astrocyte cDNA to test for PCR inhibitors. A linear relationship was seen between log-transformed template concentration and Cq value, indicating no significant PCR inhibition.

Additionally, “no RT” reactions were performed for each sample. cDNA samples were considered free from genomic DNA contamination if Cq values of “no RT” samples were at least 10 cycles higher than matched sample, or no amplification was seen within 40 cycles. All samples met this criterion. No-template control reactions were run on each plate to confirm that no exogenous DNA contamination was present. A pooled sample in which all targets were detectable at known levels was run on each plate as a positive control and to monitor inter-assay variation.

### Cell lysate preparation

For total protein preparations, cells and slices were washed once in ice-cold PBS and lysates were prepared in RIPA buffer with protease and phosphatase inhibitors. For chronic binge-drinking animals, cerebellar lysate was prepared in RIPA buffer with protease and phosphatase inhibitors from fresh tissue. All samples were sonicated for 1 min on ice and centrifuged at 14,000 rpm for 20 min. The protein concentration in the supernatant was measured using a BCA Protein Assay Kit.

### Western blotting

Cell and tissue lysates (20–50 µg total protein) were separated by SDS-PAGE electrophoresis, and protein was transferred to nitrocellulose membranes for 1.5 hours in transfer buffer with 15% methanol. Membranes were blocked for 1 hr in TBST with 2% BSA and 3% milk, then incubated with L1 antibody (1∶1000) in blocking solution for 16–18 hrs at 4°C. Blots were then incubated with HRP-conjugated anti-goat secondary antibody (1∶4000) in blocking buffer for 1 hr at room temperature. For detection of β-tubulin, anti-β-tubulin antibody (1∶000) and HRP-conjugated anti-rabbit secondary antibody (1∶4000) were used following the same protocol. Blots were either cut at 75 kD so that L1 and β-tubulin could be processed simultaneously, or membranes were stripped with Re-Blot Plus following L1 blotting and then processed for β-tubulin as described. Signals were detected with ECL Western blot substrate, and blots were then exposed to x-ray film and developed.

### Data analysis

All real time PCR data were managed and analyzed using the web-base JAVA application QPCR [67] (http://esus.genome.tugraz.at/rtpcr). The AnalyzerMiner algorithm was used to generate efficiency and Cq values for each reaction and to perform endogenous control normalization and efficiency corrections [68]. Permutation mean tests (performed in the QPCR application) were used to generate relative expression values and corresponding standard error values for each statistical class and to determine statistical significance.

For Western blotting, films were scanned and densitometry was performed using TINA 2.0 software. For each sample, L1 OD-background values were normalized to corresponding β-tubulin values and then, within each experiment, the control sample was set to 100% and all treatments were scaled accordingly. GraphPad Prism v4.0 was used to perform the one-sample *t*-test comparing the normalized means of treatment groups to 100%, the relative value assigned to control.
